# *Alpinia zerumbet* (Pers.): Food and Medicinal Plant with Potential In Vitro and In Vivo Anti-Cancer Activities

**DOI:** 10.3390/molecules24132495

**Published:** 2019-07-08

**Authors:** Maram Hussein Zahra, Tarek A.R. Salem, Bishoy El-Aarag, Nermeen Yosri, Samah EL-Ghlban, Kholoud Zaki, Amel H. Marei, Aida Abd El-Wahed, Aamer Saeed, Alfi Khatib, Mohamed F. AlAjmi, Abdulrahman M. Shathili, Jianbo Xiao, Shaden A. M. Khalifa, Hesham R. El-Seedi

**Affiliations:** 1 Division of Chemistry and Biotechnology, Graduate School of Natural Science and Technology, Okayama University, Okayama 7008530, Japan; 2 Department of Biochemistry, College of Medicine, Qassim University, Al-Qassim 51452, Saudi Arabia; 3 Department of Molecular Biology, Genetic Engineering & Biotechnology Institute, University of Sadat City, Sadat City 32958, Egypt; 4 Biochemistry Division, Chemistry Department, Faculty of Science, Menoufia University, Shebin El-Koom 32512, Egypt; 5 Department of Chemistry, Faculty of Science, Menoufia University, Shebin El-Koom 32512, Egypt; 6 Division of Pharmacognosy, Department of Medicinal Chemistry, Uppsala University, Biomedical Centre, Box 574, SE-751 23 Uppsala, Sweden; 7 Department of Bee Research, Plant Protection Research Institute, Agricultural Research Centre, Giza 12627, Egypt; 8 Department of Chemistry, Quaid-i-Azam University, Islamabad 45320, Pakistan; 9 Department of Pharmaceutical Chemistry, Faculty of Pharmacy, International Islamic University Malaysia, Kuantan 25200, Pahang, Malaysia; 10 Pharmacognosy Group, College of Pharmacy, King Saud University, Riyadh 11451, Saudi Arabia; 11 Al-Rayan Research and Innovation Center, Al-Rayan Colleges, Medina 42541, Saudi Arabia; 12 Institute of Chinese Medical Sciences, University of Macau, Taipa, Macau 999078, China; 13 Clinical Research Centre, Karolinska University Hospital, 14186 Huddinge, Sweden; 14 Department of Molecular Biosciences, The Wenner-Gren Institute, Stockholm University, SE 106 91 Stockholm, Sweden; 15 College of Food and Biological Engineering, Jiangsu University, Zhenjiang 212013, China

**Keywords:** *Alpinia zerumbet*, 5,6-dehydrokawain, Ehrlich ascites carcinoma, anti-tumor, anti-oxidant

## Abstract

Background/Aim: Plants play an important role in anti-cancer drug discovery, therefore, the current study aimed to evaluate the biological activity of *Alpinia zerumbet* (*A. zerumbet*) flowers. Methods: The phytochemical and biological criteria of *A. zerumbet* were in vitro investigated as well as in mouse xenograft model. Results: *A. zerumbet* extracts, specially CH_2_Cl_2_ and MeOH extracts, exhibited the highest potent anti-tumor activity against Ehrlich ascites carcinoma (EAC) cells. The most active CH_2_Cl_2_ extract was subjected to bioassay-guided fractionation leading to isolatation of the naturally occurring 5,6-dehydrokawain (DK) which was characterized by IR, MS, ^1^H-NMR and ^13^C-NMR. *A. zerumbet* extracts, specially MeOH and CH_2_Cl_2_ extracts, exhibited significant inhibitory activity towards tumor volume (TV). Furthermore, *A. zerumbet* extracts declined the high level of malonaldehyde (MDA) as well as elevated the levels of superoxide dismutase (SOD) and catalase (CAT) in liver tissue homogenate. Moreover, DK showed anti-proliferative action on different human cancer cell lines. The recorded IC_50_ values against breast carcinoma (MCF-7), liver carcinoma (Hep-G2) and larynx carcinoma cells (HEP-2) were 3.08, 6.8, and 8.7 µg/mL, respectively. Conclusion: Taken together, these findings open the door for further investigations in order to explore the potential medicinal properties of *A. zerumbet.*

## 1. Introduction

Cancer is a complex group of diseases that is characterized by a rapid and uncontrolled formation of abnormal cells, which may collect together to form a tumor. The development of cancer occurs through a multistage process and driven by endogenous and environmental factors [1,2,3,4]. Oxidative stress is the imbalance between the anti-oxidant enzymes production included superoxide dismutase (SOD), catalase (CAT), and glutathione peroxidase (GPx) and the release of free radicals in the metabolic or phagocytic process [5]. Oxidative stress causes damage to the DNA molecule, modifies signaling pathways, and regulates progression of various types of cancers [6]. Cancer initiation and progression has been combined to oxidative stress through increasing DNA mutations and damage, breakage of genome structure, and induction of cell proliferation [7]. Due to the drawbacks of chemotherapeutic treatment [8], medicinal plants and venoms play important roles in anti-cancer drug discovery [9,10,11]. 

*Alpinia* species are used as human food components with pharmaceutical effects in many countries. In Asia is used to relieve fevers and malaria, as well as to act as general health improvements [12]. They are important natural resources for their use as spice crops [13]. In Japan, it is used to prepare a traditional food, mu-chi, that prevents the common cold [14]. In China, it is employed to clear cold and stimulate the spleen and stomach as well as in Brazil it is usually used as a drink to treat hypertension and as water pill medication [15,16]. *A. zerumbet* (Zingiberaceae) species is originating in the East Indies and is widespread in the tropical and subtropical regions of South America, Oceania, and Asia [17]. It is used in traditional medicine to cure cardiovascular disorders, hypertension, inflammation, cold, and as antispasmodic agent. Also, the essential oil possess anti-oxidant, relaxant, anti-spasmodic and anti-cancer effects [18,19]. Moreover, *A. zerumbet* extract possessed anti-nociceptive, anti-pyretic, and anti-inflammatory activites mediated by free radicals scavenging and inhibition of prostaglandins and leukotrienes synthesis [20]. 

As part of the current ongoing research efforts, we wanted to exploit the opportunity of finding a new potential anti-cancer entity using the unique set of Egyptian medicinal plants that are traditionally used by the Bedouins of the Sinai desert. These plants have not been chemically investigated and are known to have biological activities, which maximizes the chance of providing novel molecular structures with promising cytotoxic and anti-oxidant effects. In this particular study we focused on investigating the chemical components of the active fractions of *A. zerumbet* flowers. Also, the active fractions were subjected to bioassay-guided protocol, developed for in vitro and in vivo studies prior to the isolation of the bioactive ingredients [21]. The present study revealed that *A. zerumbet* extracts possessed significant suppression effect against solid tumor volume. DK showed anti-proliferative effect against different human cancer cell lines. In addition, the levels of MDA, SOD, and CAT in liver tissue homogenates were regulated by the action of extracts. 

## 2. Results

### 2.1. Cytotoxicity of Methanol Extracts of Fifteen Egyptian Medicinal Plants Species

The cytotoxicity of methanol (MeOH) extracts of Egyptian medicinal plants species was determined using brine shrimp (*Artemia salina*). Results revealed that the methanol extracts of the species possessed variable range of mortality against brine shrimp. Mortality percent of the fifteen plant species that were evaluated in this screening were listed in Table 1. The flowers of A. *zerumbet* exhibited the most potent activity among all the tested methanol extracts, with mortality rate of 93.33%. 

### 2.2. Cytotoxicity of A. zerumbet Extracts/Fractions

Since the MeOH extract of *A. zerumbet* showed the highest potent activity, bioassay-guided fractionation was performed to isolate these cytotoxic bioactive compounds. *A. zerumbet* extract was successively partitioned by hexane, CH_2_Cl_2_ and H_2_O. The bioactivity of *A. zerumbet* extracts/fractions was detected using brine shrimp lethality. Results in Table 2 represented that the most significant activity was displayed particularly in CH_2_Cl_2_ with mortality rate (100%). Also, the Bioassay-guided fractionation resulted in the isolation of 5,6-dehydrokawain (DK).

### 2.3. Structure Elucidation of DK

**Figure 1:**
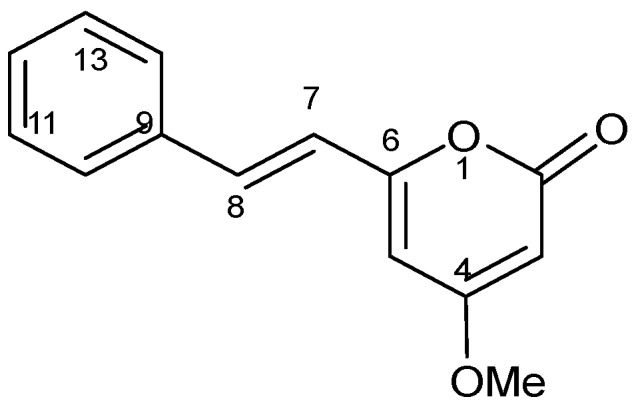
Chemical structure of 5,6-dehydrokawain (DK).

To find out the molecular structure of the active main compounds, CH_2_Cl_2_ extract was fractionated using SEPARO-AGC, followed by bio-assay. Fraction 1 possessed the strongest activity causing brine shrimp mortality of 100% (Table 2), therefore, the compound was isolated from fraction 1 as pale yellow crystals with a molecular formula of C_14_H_12_O_3_, which was established by the EIMS (*m*/*z* 228 Da). Using spectroscopic analysis, DK was identified as shown in Table 3 and chemical structure was illustrated in Figure 1.

### 2.4. In Vitro Cytotoxic Activity of A. zerumbet Extracts Against Ehrlich Ascities Carcinoma (EAC) Cells

**Figure 2:**
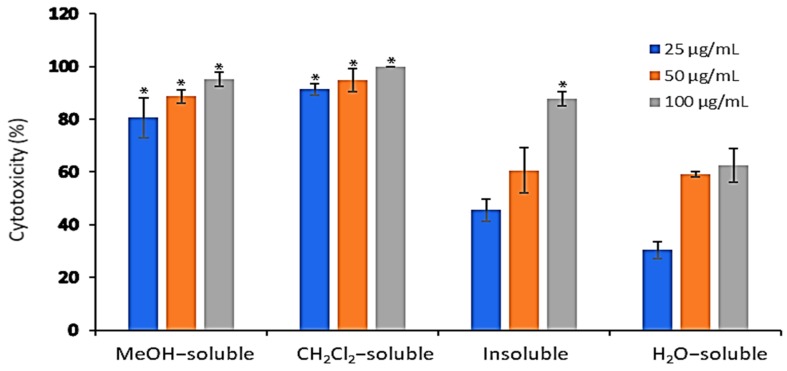
The in vitro cytotoxic effect of *A. zerumbet* extracts. The cytotoxicity of *A. zerumbet* extracts (MeOH, CH_2_Cl_2_, insoluble and H_2_O) was evaluated against EAC cells using trypan blue exclusion assay. Three concentrations (25, 50, and 100 µg/mL) were added on the cells in triplicate and incubated at 37 °C for 3 h in 5% CO_2_ atmosphere. Data was expressed as mean ± SEM from three independent experiments. Significantly (* *p* < 0.001) different from the vehicle control (DMSO).

The cytotoxic activity of *A. zerumbet* extracts (crude MeOH, CH_2_Cl_2_, insoluble and H_2_O) was evaluated against EAC cells at three concentrations (25, 50, and 100 µg/mL) using trypan blue exclusion assay. Results in Figure 2 showed that the cytotoxic activity of the tested extracts was concentration dependent manner. The recorded activities of MeOH, CH_2_Cl_2,_ insoluble and H_2_O extracts at 25 µg/mL were 80.5%, 91.3%, 45.5% and 30.5%, respectively, while at 50 µg/mL were 88.6%, 94.7%, 60.6%, and 59%, respectively. The highest cytotoxic activities were observed at 100 µg/mL as followed 95.2%, 100%, 87.8% and 62.5%, respectively compared to that of vehicle control, dimethylsulfoxide (DMSO).

### 2.5. Anti-Proliferative Activity of DK

**Figure 3:**
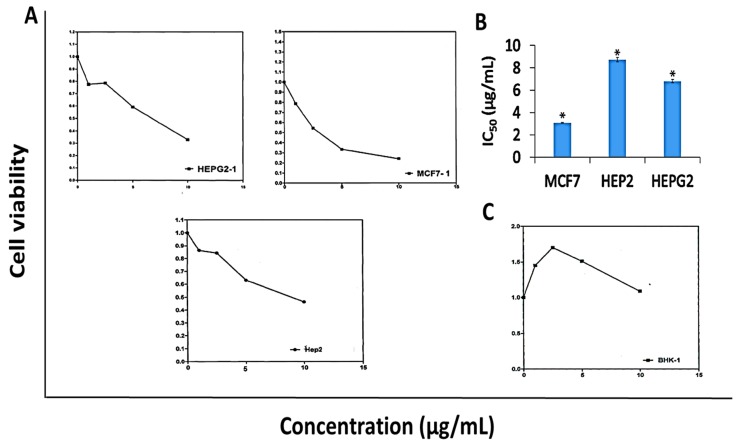
Anti-proliferative activity of 5,6-dehydrokawain (DK) against MCF-7, HEP-G2, HEP-2, and BHK cell lines. Cell viability was evaluated using MTT assay. (**A**) Dose-response curves, (**B**) IC_50_ values of DK against MCF-7, HEP-G2, and HEP-2, (**C**) Effect of DK against human normal fibroblast (BHK). IC_50_ values were calculated from dose-response curves and represented as mean ± SME of three independent experiments. Significantly (* *p* < 0.001) different from the vehicle control (DMSO).

Results in Figure 3 revealed that DK showed anti-proliferative activity against MCF-7, HEP-G2, and HEP-2 cell lines (Figure 3A) with calculated IC_50_ values of 3.08, 6.8, and 8.7 µg/mL, respectively as illustrated in Figure 3B. Conversely, DK didn’t show cytotoxic activity against normal human fibroblast cell line (BHK) at the concentration range (1–10 µg/mL) as shown in Figure 3C.

### 2.6. Effect of A. zerumbet Extracts on Tumor Volume (TV)

**Figure 4:**
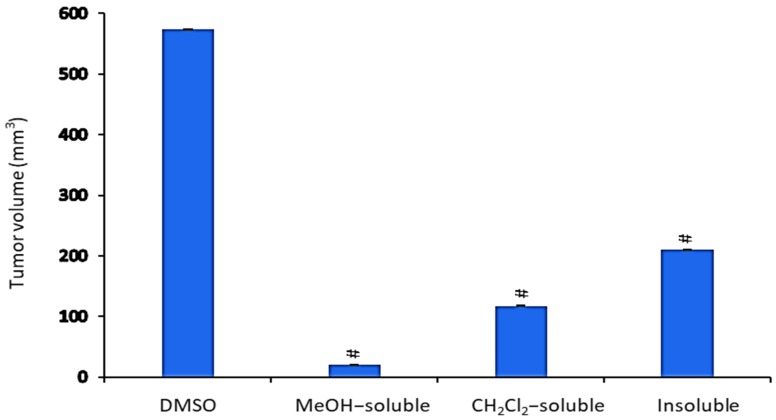
The effect of *A. zerumbet* extracts (MeOH, CH_2_Cl_2_, and insoluble) on tumor volume (TV). TV was calculated from the current equation: TV(mm^3^) = 0.52 × longest diameter × (shorter diameter)^2^. Data was expressed as mean ± SEM. Significantly (^#^ *p* < 0.001) different from the vehicle control (tumor + DMSO).

All *A. zerumbet* extracts showed a significant (*p* < 0.001) reduction in TV in comparison to the control group (Figure 4). MeOH extract exhibited the most potent inhibitory effect (96.6%) followed by CH_2_Cl_2_ and insoluble extracts (79.6% and 63.2%, respectively) as compared to that of vehicle control (tumor + DMSO).

### 2.7. Effect of A. zerumbet Extracts on Malonaldehyde (MDA) Level

**Figure 5:**
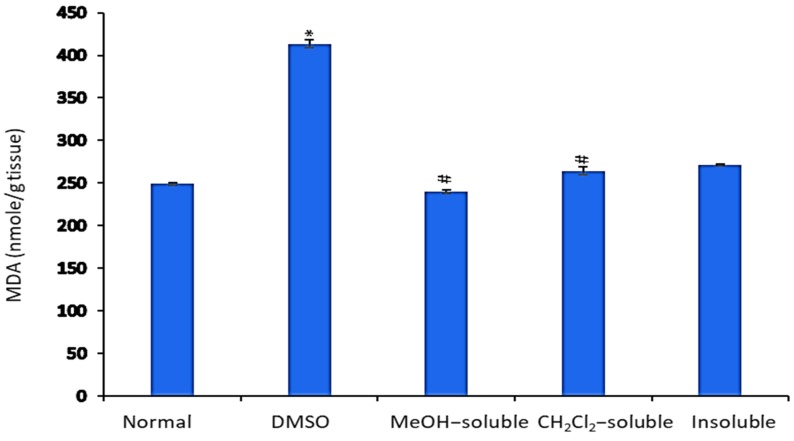
Effect of *A. zerumbet* extracts (MeOH, CH_2_Cl_2_, and insoluble) on malonaldehyde (MDA) activity. MDA is an indicator of lipid peroxidation. MDA levels were assessed in liver homogenate, data was represented as mean ± SEM and expressed as nmole/g tissue. Significantly (* *p* < 0.001) different from the normal control (untreated) and Significantly (^#^ *p* < 0.001) different from the vehicle control (tumor + DMSO).

The levels of MDA in the liver tissue homogenate were measured as an indicator of lipid peroxidation. As shown in Figure 5, MDA levels were significantly (*p* < 0.001) increased in mice bearning solid tumor compared to those in the normal control group (untreatred). In contrast, EAC-bearning mice treated with crude MeOH, CH_2_Cl_2_, and insoluble extracts exhibited a significant (*p* < 0.001) reduction in MDA level compared to that of vehicle control group (tumor + DMSO). 

### 2.8. Effect of A. zerumbet Extracts on Superoxide Dismutase (SOD) Activity

**Figure 6:**
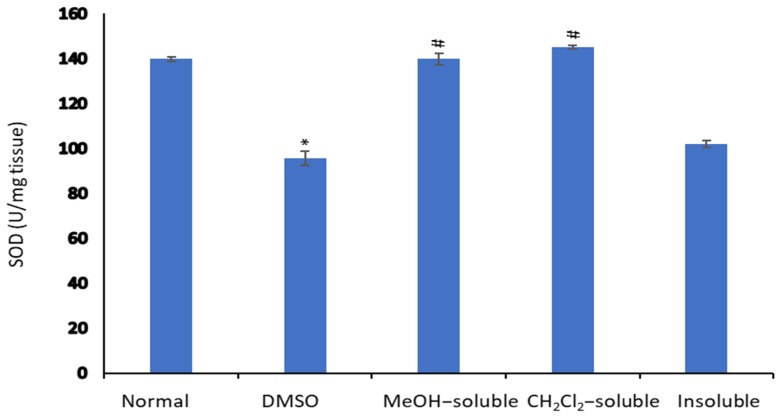
Effect of *A. zerumbet* extracts (MeOH, CH_2_Cl_2_, and insoluble) on superoxide dismutase (SOD) activity. SOD levels were assessed in liver homogenate, data was represented as mean ± SEM and expressed as U/mg tissue. Significantly (* *p* < 0.001) different from the normal control (untreated) and significantly (^#^ *p* < 0.001) different from the vehicle control (tumor + DMSO).

Figure 6 illustrated that the level of SOD in the liver tissue homogenate was significantly declined (*p* < 0.001) in tumor-bearing mice compared to normal control mice (untreated). On the other hand, the treatment of mice bearning solid tumor with MeOH, CH_2_Cl_2_ and insoluble extracts resulted in significant elevation (*p* < 0.001) and restoring the SOD level compared to that of vehicle control (tumor + DMSO). 

### 2.9. Effect of A. zerumbet Extracts on Catalase (CAT) Activity

**Figure 7:**
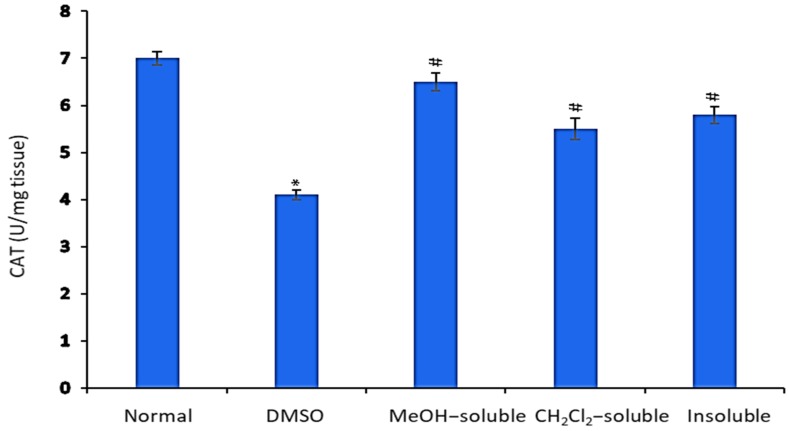
Effect of *A. zerumbet* extracts (MeOH, CH_2_Cl_2_, and insoluble) on catalase (CAT) activity. CAT levels were assessed in liver homogenate, data was represented as mean ± SEM and expressed as U/mg tissue. Significantly (* *p* < 0.001) different from the normal control (untreated) and significantly (^#^ *p* < 0.001) different from the vehicle control (tumor + DMSO).

In comparison to normal control mice (untreated), the level of CAT in the liver tissue homogenate was significantly declined (*p* < 0.001) in tumor-bearing mice as shown in Figure 7. Conversely, the mice treated with MeOH, CH_2_Cl_2_ and insoluble extracts showed a significant (*p* < 0.001) elevation in CAT level in relative to that of vehicle control group (tumor + DMSO). 

## 3. Discussion

Cancer is a leading cause of cancer-related deaths worldwide [26]. Surgery, radiation, and chemotherapy are methods used for cancer treatment. Although many synthetic anti-cancer agents are used in the treatment of cancer, the side effects and drug resistant still limited their use [27]. Therefore, anti-cancer agents from natural products, particularly medicinal plants are desirable [28]. Medicinal plants such as polyphenolic compounds and dietary polyphenols exhibited beneficial effects in human health. As, they can induce prevention of cancers, cardiovascular and neurodegenerative diseases [29]. Several herbs possessing cancer-preventive properties has identified through the National Cancer Institute [30]. 

In the current study fifteen Egyptian medicinal plants species (Table 1) have chosen since their tested parts are known to contain polyphenolic compounds as well as different active ingredients that may exhibited several biological functions. Table 1 revealed that the methanol extract of *A. zerumbet* flowers exhibited the most potent activity among the studied fifteen Egyptian medicinal plants extracts. The bioassay-guided fractionation for *A. zerumbet* extract was performed by hexane, CH_2_Cl_2_ and H_2_O and according to results in Table 2, CH_2_Cl_2_ extract exhibited the most significant activity. The bioassay-guided fractionation of CH_2_Cl_2_ extract leaded to the isolation of DK (Figure 1) and its chemical structure was elucidated by different spectroscopic methods (Table 3). Moreover, DK showed anti-proliferative activity against MCF-7, HEP-G2, and HEP-2 as shown in Figure 3.

EAC tumor was primarily described as a spontaneous murine mammary adenocarcinoma [31] with very aggressive growing in mice. Its ascites form has been used as a tumor model to examine the anti-tumor effects of several substances [32]. Therefore, the cytotoxic activity of different *A. zerumbet* extracts was evaluated against EAC cells. Results in Figure 2 showed that all extracts proven in vitro cytotoxic activity specially, CH_2_Cl_2_ extract. Additionally, they possessed potent activity to reduce the tumor mass in a mouse xenograft model according to results of Figure 4. This may be explained and related to the presence of cytotoxic active compound, DK. 

Under metabolic pathways and certain conditions, oxygen molecule generated undesirable reactive oxygen species (ROS) such as superoxide anion and hydroxyl radicals as well as non-free radical species as hydrogen peroxide. The excessive production of free radicals reported to induce lipid peroxidation in vivo [33,34]. The MDA, the end product of lipid peroxidation, found to be a tumor promoter which caused high toxicity and inhibition of some protective enzymes [35,36]. The excessive amount of ROS can lead to many several diseases, including cancer [37]. Thus, the anti-oxidant effects of the *A. zerumbet* extract in liver homogenate of mice bearing EAC-solid tumor was evaluated.

As shown in Figure 5, in EAC bearing mice, the level of lipid peroxide in liver was significantly elevated, which was however reduced near to normal level in *A. zerumbet* extract treated mice. This reflects the decline in free radical production and subsequent reduction in oxidative stress. MDA found to be high in cancer tissues [38], which is compatible with our present results in untreated EAC control groups. Moreover, several studies demonstrated the anti-lipid peroxidative action of *A. zerumbet* [39,40,41], which is in agreement with our present results.

Enzymatic anti-oxidant mechanisms play a vital role in the removal of free radicals. SOD reported as the first line of defense against superoxide anion and preventing its destructive effect. It is involved in the clearance of superoxide anions. The inhibition of SOD activities as a result of tumor growth was reported [42]. Results in Figure 6 showed a significant decrease in SOD level in EAC-bearing mice when compared with treated groups, which is in agreement with other reported studies [43,44]. The administration of *A. zerumbet* significantly recovered the SOD level towards normal level. 

The free radical scavenging enzyme, CAT, is existing in all oxygen-metabolizing cells and acts as a direct defense against the potentially damaging reactivities of superoxide and hydrogen peroxide. The inhibition of CAT activities as a result of tumor growth has also been reported [31]. According to results illustrated in Figure 7, EAC tumor-bearing mice exhibited a significant decrease in CAT, which is in agreement with that previously reported [45]. The administration of *A. zerumbet* increased the CAT levels, which along with the restoration of lipid peroxide contents to near normal level indicate the anti-oxidant and free radical scavenging property of *A. zerumbet* and its potential use as anti-cancer agent.

## 4. Materials and Methods

### 4.1. General Experimental Procedures

TLC was done on silica 60 F_254_ plates (0.25 mm; Merck, Munich, Germany), and detected with UV light (254 and 366 nm). The melting point was measured using a Digital Melting Apparatus (Model 1A 8103, Electrothermal Engineering Ltd., London, UK); IR spectrum was recorded using a Perkin-Elmer 1600 FTIR spectrometer (Washington, WA, USA). HPLC/ESI-MS chromatograms and spectra for dereplication were obtained on a Finnigan LCQ (San Jose, CA, USA) ion-trap mass spectrometer coupled with an electro spray interface, and ÄKTA basic 10 HPLC systems (Amersham Biosciences, Uppsala, Sweden). For LC-DAD-fractionation, a Shimadzu LC-10 system, equipped with an SPD-M10AVP diode array detector, was employed. UV data were collected at 190 and 600 nm. It is composed of a P-900 pump, UV-900 detector, Frac-900 fraction collector, with monitors at wavelengths of 217, 254, and 335 nm. Accelerating gradient chromatography (AGC) was performed using variable length AGC glass columns (Baeckström Separo AB, Lidingö, Sweden) with inner diameters of 4.00, 2.50, and 1.50 cm, packed with silica gel 60, 40–63 µm (Merck) and an FMI Lab pump, model QD (Fluid Metering Inc., Oyster Bay, NY, USA), delivering a flow rate of 15–18 mL/min. Sample was eluted by a continuous gradient flow running from hexane, through CH_2_Cl_2_, EtOAc, and MeOH to H_2_O. NMR spectra were recorded on a Bruker DRX 600 spectrometer at 600.1 and at 150.9 MHz, respectively at 25 °C using CDCl_3_ as the solvent. All chemical shifts are expressed relative to TMS [46,47].

### 4.2. Plants Material 

Plant specimens listed in (Table 1) were collected from the North, South Sinai, El-Menoufia and different areas of Egypt between 2006 and 2009. The specimens were identified by Prof. Loutfy Boulos (Department of Botany, Faculty of Science, Alexandria University, Alexandria, Egypt), and Prof. Zaki Turki (Department of Botany, Faculty of Science, Menoufia University, Egypt). Voucher specimens were deposited in the herbarium of the Department of Botany in the Faculty of Science, Menoufia University, Egypt.

### 4.3. Extraction and Isolation

Plant materials were cut into small pieces and dried at room temperature. The dried material was grounded to a coarse powder and extracted using MeOH (1 week) at room temperature three times in succession with occasional stirring. The extracts were filtered and evaporated under reduced pressure. All extracts were submitted to the biological assays, and based on initial screening; A. zerumbet was selected for further analysis. A. zerumbet (300 g) was extracted three times (one week each) with MeOH to give 34 g then partitioned between n-hexane, CH_2_Cl_2,_ and H_2_O to give 4.9, 6, and 19.5 g, respectively. CH_2_Cl_2_ extract (6 g) was adsorbed onto silica gel (12 g) and chromatographed on a silica gel (28 g) column, eluted with continuous hexane-CH_2_Cl_2_, CH_2_Cl_2_-MeOH to MeOH gradients. Five major fractions (F1:F5) were obtained. F1 was subjected to dereplication, HPLC fractionation, and LC/ESI-MS to yield the pure compound (6 mg). The amount of the compound was 0.002 g for each 100 g of the dried plant.

### 4.4. Detection of the Cytotoxicity of Egyptian Medicinal Plants 

The cytotoxicity of MeOH extracts of fifteen medicinal plants species and A. zerumbet extract were assessed using brine shrimp lethality bioassay [48]. The extracts were examined in triplicate at 100 µg/mL in vials containing 5 mL of brine and 10 shrimp. The number of surviving shrimp after 24 h was counted and the mortality percentage was calculated as the follows % of death = [(test-control) /control] × 100. LD_50_ was derived from the best fit line obtained by linear regression analysis [49].

### 4.5. Determination of the Cytotoxicity of A. zerumbet Extracts

The cytotoxic effect of crude MeOH, CH_2_Cl_2_, insoluble and H_2_O extracts of *A. zerumbet* were determined in vitro against EAC cells using trypan blue dye exclusion method [50]. Cells viability were assayed in the presence of three concentrations (25, 50, and 100 µg/mL). In details, EAC cells aspirated aseptically from the peritoneal cavity of the mice and washed with Hank’s balanced salt solution (HBSS) and centrifuged for 15 min at 1,500 rpm in a cooling centrifuge. The pellet re-suspended with HBSS and the process repeated three times. Finally, the cells were suspended in known volume of RPMI-1640, supplemented with 10% fetal bovine serum, 10 µg/mL streptomycin and 4 µg/mL penicillin; the cell count adjusted to 2 × 10^6^ cells/mL. Then, 0.2 mL of this diluted cell suspension titrated in 96 flat-bottomed tissue culture plates. Three concentrations (25, 50, and 100 µg/mL) were added on the cells in triplicate and incubated at 37 °C for 3 h in 5% CO_2_ atmosphere. The total number of dead and living cells was counted using a hemocytometer and cell viability/cytotoxicity percentage was calculated [51].

### 4.6. Anti-Proliferative Activity of DK

#### 4.6.1. Cell Lines and Cells Culture

HEP-G2 (human liver carcinoma), MCF-7 (humn breast carcinoma), HEP-2 (human larynx carcinoma), and BHK (human normal fibroblast) were cultured in RPMI medium (Sigma-Aldrich, St. Louis, MO, USA) supplemented with 10% fetal bovine serum (FBS, PAA Laboratories, Pasching, Austria) in the presence of 100 IU/mL penicillin and 100 μg/mL streptomycin. The cells were maintained at 37 °C in a humidified incubator with 5% CO_2_. Culture media were changed every two days with new ones.

#### 4.6.2. Proliferation Assay

The anti-proliferative activity of DK was determined using 3-(4,5-dimethylthiazol-2-yl)-2,5-diphenyltetrazolium bromide (MTT) colorimetric assay. Cells were seeded at density of 5 × 10^3^ cells/well in 96-well plates. After 24 h from seeding, the media were changed with new culture media containg different concentration of DK (1–10 µg/mL). Post 24 h of DK addition, MTT (5 µL) were added to the cells for 4 h. The formed formazan crystals were dissolved by adding 10% SDS containing 0.02 N HCl for 24 h and then the absorbance was measured at 570 nm. Percentage of viability was calculated compared to vehicle control (DMSO) and the half maximal growth inhibitory concentration (IC_50_) was determined from the line equation of the dose-response curve.

### 4.7. In Vivo Studies

#### 4.7.1. Tumor Cells 

EAC cells were supplied by the National Cancer Institute, Cairo, Egypt. The cells were maintained through interperitoneal (i.p) transplantation of 1 × 10^6^ viable tumor cells in 1 mL of saline in normal healthy mice by using 25G needle, to be used later for in vivo experiments.

#### 4.7.2. Animals 

Adult female Swiss albino mice (6–7 weeks old) with an average body weight of 25 g were used in the present study. Mice were acclimatized for 7 days under standard laboratory conditions and fed with libitum and water before commencement of the experimental protocol. The present study protocol was approved by the Ethical Committee for Laboratory Animals of Science Faculty Menoufia University (No.: ECLA-SFMU-10217). 

#### 4.7.3. Mouse Xenograft Model

Solid tumors were induced in mice by subcutaneous injection of 0.2 mL of EAC cells (2 × 10^6^ cell/mL) [52]. Seven days after inoculation, the mice were randomly divided into 4 groups. (1) MeOH group, (2) CH_2_CL_2_ group, (3) insoluble extract group and (4) control group: Groups 1, 2, and 3 were treated subcutaneously with 100 µL of 250 mg/kg from MeOH, CH_2_Cl_2_, and insoluble extracts, respectively daily for 5 consecutive days. The control group was treated with extracts vehicle (0.03% DMSO). After treatment period, mice were euthanized then solid tumors and liver tissues were dissected. The tumor volume (TV) was calculated using the following formula TV(mm^3^) = 0.52 × longest diameter × (shorter diameter)^2^ [53]. Liver tissue homogenate, (10% (*w*/*v*)) in potassium phosphate buffer pH 7.4, were used for detection of MDA and SOD, CAT levels.

#### 4.7.4. Estimation of MDA Levels in Liver Homogenate

MDA levels are an index of lipid peroxidation and were determined in the liver tissue homogenate according to a previously reported method [54]. Briefly, thiobarbituric acid reacts with MDA in the homogenate to form a thiobarbituric acid reactive product. The coloured product was measured at 534 nm and the results were expressed as nmol/g tissue.

#### 4.7.5. Estimation of SOD Levels in Liver Homogenate

SOD, metalloenzyme, catalyzes the dismutation of the superoxide anion to molecular oxygen and hydrogen peroxide. SOD activity was measured according to the Beyer method [55] and expressed as U/mg tissue.

#### 4.7.6. Estimation of CAT Levels in Liver Homogenate

CAT was determined in the liver tissue homogenate through the method of Aebi [56]. Briefly, CAT reacted with a known quantity of H_2_O_2_ and after one minute the reaction was stopped. The remaining H_2_O_2_ reacts with 4-aminophenazone to form a coloured product. CAT activity was measured and expressed as U/mg tissue.

### 4.8. Statistical Analysis

Data are expressed as mean ± SEM and the statistical comparison between groups were analyzed using one-way ANOVA followed by Tukey post hoc test (Graph Pad Software version 6, San Diego, CA, USA). A *p* of < 0.05 value was considered statistically significant.

## 5. Conclusions

Our study revealed that *A. zerumbet* flowers were the most potent plant among the fifteen Egyptian medicinal plants. *A. zerumbet* extracts showed potent anti-tumor effects against EAC. DK was isolated from CH_2_Cl_2_ extract and characterized by different microscopic techniques. Additionally, *A. zerumbet* extracts exhibited significant inhibitory activity towards TV as well as restored the levels of MDA, SOD, and CAT in liver tissue homogenates. DK exhibited anti-proliferative activity against different human cancer cell lines. Further investigations were required to explore the potential medicinal mechanisms and clinical application of *A. zerumbet*.

## Figures and Tables

**Table 1:** Lethality of Egyptian medicinal plants’ MeOH extracts using brine shrimp (*Artemia salina*).

No.	Plant Scientific Name	Vernacular Name	Part Used	% Mortality
1	*Alpinia zerumbet*	Variegated ginger	Flowers	93.33
2	*Anabasis setifera*	Agram, Asal, Glew عجرم, عسل، جلو	Flowers	43.33
3	*Ballota undulata*	Ghassa, Zafra غصة، زفرة	Flowers	50.00
4	*Calathea metallica*	ND	Flowers	23.33
5	*Caylusea hexagyna*	Danaban دنبان	Flowers	10.00
6	*Echinops glaberrimus*	Khashir خشير	Flowers	56.66
7	*E. spinosus*	Qatad, Gorreih قتاد، جريح	Flowers	86.66
8	*Globularia arabica*	Handaqouq, zorreiqa حندوق، زُريقة	Flowers	46.66
9	*Lavandula pubescens*	Atan عطن	Fruits	63.33
10	*Psoralea mutisii*	ND	Flowers	13.33
11	*P. pubescens*	ND	Flowers	6.66
12	*Reseda arabica*	ND	Flowers	30.00
13	*Salvadora persica*	Araak, Siwaak, Miswaak أراك، سواك، مسواك	Flowers	0.00
14	*Salvia aegyptiaca*	Ra‘alah رعلة	Flowers	63.33
15	*Senecio reflexun*	Morrar, Umm Lonein مررار, أم لونين	Flowers	36.66

**Table 2:** Bioactivity evaluation of *A. zerumbet* extracts/fractions using brine shrimp lethality.

Substance	Mortality %
	Concentration 100 (µg/mL)
CH_2_Cl_2_ ex.	100.00
MeOH-H_2_O (90%) ex.	80.00
H_2_O ex.	50.00
Hexane ex.	50.00
Insoluble ex.	13.33
F1	100.00
F2	30.00
F3	20.00
F4	63.33
F5	86.66
DK	100.00

ex.: extract; F: fraction; DK: 5,6-dehydrokawain.

**Table 3:** ^1^H and ^13^C-NMR data using CDCl_3_ solvent.

Position	Type	δH(ppm)	δc(ppm)	References
2	C	−	162.9	
3	CH	5.3,s	88.6	[22]
4	C	−	170.7	
5	CH	5.87,s	101	[23]
6	C	−	158.6	
7	CH	6.41,d	118.7	[24]
8	CH	7.39,d	135.4	
9	C	−	135.3	[25]
10	CH	7.29,d	127.1	
11	CH	7.29,dd	128.6	
12	CH	7.29,dd	129.1	
13	CH	7.29,dd	128.4	
14	CH	7.29,dd	127.1	
4-OMe	CH_3_	3.77,s	55.7	
